# Virtual Screening of Marine Natural Products Targeting the F Protein for Anti-RSV Drug Discovery

**DOI:** 10.3390/ijms27052484

**Published:** 2026-03-08

**Authors:** Wenqing Liu, Xuran Gu, Ruikun Du, Zhiqing Liu, Pingyuan Wang, Chang-Yun Wang

**Affiliations:** 1 MOE Key Laboratory of Marine Drugs and Key Laboratory of Evolution and Marine Biodiversity, Institute of Evolution & Marine Biodiversity, School of Medicine and Pharmacy, Ocean University of China, Qingdao 266003, China; lwq2089@stu.ouc.edu.cn (W.L.); liuzhiqing@ouc.edu.cn (Z.L.); 2 Laboratory for Marine Drugs and Bioproducts, Qingdao Marine Science and Technology Center, Qingdao 266237, China; 3 Innovative Institute of Chinese Medicine and Pharmacy, Shandong University of Traditional Chinese Medicine, Jinan 250355, China; guxuran0605@outlook.com (X.G.); ruikun@sdutcm.edu.cn (R.D.); 4 Qingdao Academy of Chinese Medical Sciences, Shandong University of Traditional Chinese Medicine, Qingdao 266114, China

**Keywords:** respiratory syncytial virus (RSV), fusion (F) protein, marine natural products, virtual screening, molecular docking, ADMET prediction, MM/GBSA, manzamine alkaloids

## Abstract

Respiratory syncytial virus (RSV) poses a substantial global health burden, particularly in infants and the elderly. The fusion (F) protein is a key therapeutic target for inhibiting RSV entry. In this study, we performed a structure-based virtual screening of the Comprehensive Marine Natural Products Database (CMNPD) to discover novel anti-RSV agents targeting the prefusion F protein trimer. Screening of 31,561 compounds via molecular docking, followed by stringent ADMET (absorption, distribution, metabolism, excretion, and toxicity) profiling and MM/GBSA (Molecular Mechanics/Generalized Born Surface Area) binding free energy calculations, identified 11 promising candidates. Among these, manzamine alkaloids exhibited the most favorable docking scores (as low as −13.3 kcal/mol) and promising Ligand Efficiency (LE) values. These molecules primarily interact with conserved hydrophobic residues (Phe140, Phe488) through hydrophobic interactions, π-stacking, and electrostatic forces. Our study highlights marine natural products, especially manzamine alkaloids, as promising leads for the development of novel, orally bioavailable RSV fusion inhibitors, potentially offering avenues to overcome existing drug resistance. However, these computational findings require in vitro validation to confirm efficacy.

## 1. Introduction

Respiratory syncytial virus (RSV) is a highly seasonal pathogen that primarily infects the respiratory tract and exhibits strong pathogenicity, frequently causing severe illnesses such as pneumonia and bronchiolitis [1]. While most infections are mild and self-limiting, the virus can be severe in young children, immunocompromised adults, and the elderly, potentially progressing to fatal respiratory failure [2,3]. Given its profound impact, RSV is recognized as a major public health concern, necessitating effective preventive and therapeutic strategies [4].

Currently approved clinical therapeutics for RSV primarily consist of prophylactic drugs such as monoclonal antibodies, including monoclonal antibody therapies such as Nirsevimab (Beyfortus) and Clesrovimab (Enflonsia) [5,6], and RSV vaccines like GSK’s Arexvy, Pfizer’s Abrysvo and Moderna’s mRNA vaccine mRESVIA (mRNA-1345) [7]. Their utility is primarily prophylactic, preventing infection initiation, but often ineffective against established infections. Hence, the development of small-molecular drugs remains among the key priorities for anti-RSV agent development [8].

**Figure 1:**
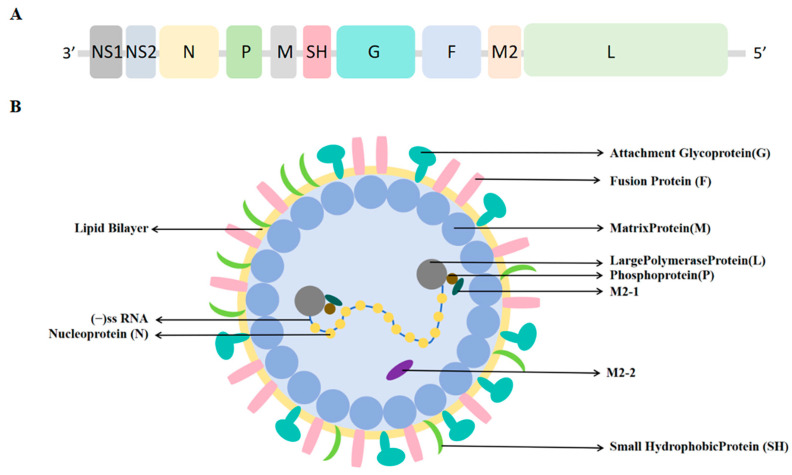
Structure of RSV. (**A**) RSV genome. (**B**) RSV virion structure. Adapted from Ref. [11] with modifications.

RSV is a non-segmented, negative-sense, single-stranded, and enveloped RNA virus [9]. Its genome encodes 11 proteins, including the attachment glycoprotein (G), fusion protein (F), and small hydrophobic protein (SH) in the envelope, as well as internal structural (N, P, L, M2-1, M2-2) and non-structural (NS1, NS2) proteins (Figure 1) [10]. Viral entry is mediated by G protein attachment and F protein-driven, clathrin-dependent endocytosis [11].

**Figure 2:**
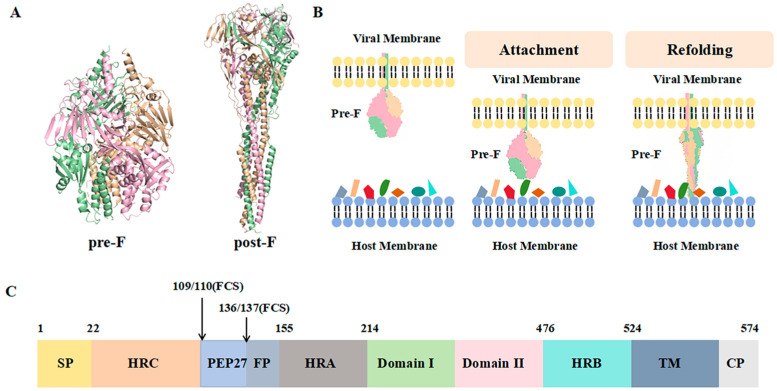
Structure of the F protein and its conformational changes before and after fusion. (**A**) A ribbon diagram illustrating the pre-F and post-F protein, both of which are trimeric. (**B**) The F protein undergoes conformational changes before and after membrane fusion. The colored elements on the host cell membrane represent attachment factors and functional receptors. Adapted from Ref. [14] with modifications. (**C**) Schematic diagram of the RSV F protein structure.

The F protein (Figure 2) represents one of the most promising therapeutic targets for anti-RSV drug design. Significant progress has been made in recent years toward elucidating the binding mode and inhibitory mechanism of fusion inhibitors targeting the F protein [12]. F protein inhibitors block viral infection by preventing the fusion of the viral envelope with the host cell membrane. The F protein exists in two distinct conformational states: the prefusion conformation (pre-F) and the postfusion conformation (post-F) (Figure 2A) [13]. The pre-F conformation represents a high-energy, metastable state that readily undergoes structural rearrangement to transition into the more stable, low-energy post-F conformation (Figure 2B) [14]. This critical step is mediated by the six-helix bundle domain of the F protein, a fusion mechanism characteristic of the Paramyxoviridae family. To date, most RSV F inhibitors developed for therapeutic purposes target the pocket deeply embedded within the central cavity of the pre-F trimer [15], thereby preventing transition to the postfusion state and the formation of its characteristic six-helix bundle. Before virus entry, the F protein trimer is maintained in the prefusion conformation, with the fusion peptide deeply buried in the protein interior and inaccessible to solvents. After a series of conformational changes, the F protein’s fusion peptide is exposed and inserted into the target cell membrane. Subsequently, the HRB and HRA domains interact to form a stable six-helix bundle (6-HB) core structure, resulting in membrane apposition. Finally, the fusion pore opens, allowing for the entry of the viral genetic material into the target cell [16].

**Figure 3:**
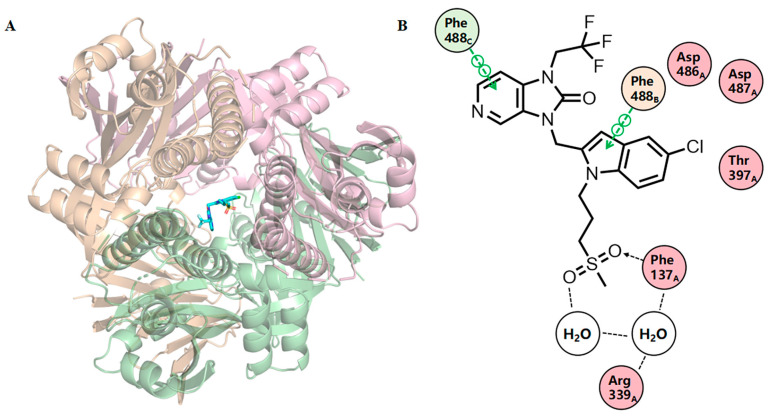
Rilematovir binds to a threefold-symmetric cavity in F protein (PDB code: 5KWW). Important residues are drawn as sticks. The ligand is shown with atom-specific coloring (C: light blue, N: dark blue, O: red, S: yellow). (**A**) Views of the crystal structure of Rilematovir bound to a pre-F trimer. The three protein monomers are depicted in distinct colors (orange, green, deep pink). (**B**) A 2D ligand-interaction diagram generated in Molecular Operating Environment. Green lines with circular nodes indicate π-stacking interactions, dashed lines represent water-mediated hydrogen bonds; dashed lines with arrowheads indicate direct hydrogen bonds (arrowheads point to the acceptors).

The most advanced F protein inhibitor, Rilematovir (JNJ-53718678), exhibits direct binding to pre-F [17]. It progressed to phase 3 clinical trials following the demonstration of potent in vitro activity (EC_50_ = 0.5 nM against recombinant rgRSV224 in HeLa cells). However, its clinical development has been terminated. The co-crystal structure revealed that Rilematovir asymmetrically occupies the binding pocket (Figure 3A) and engages in aromatic π-stacking interactions with Phe140 and Phe488 residues of the F protein. In the binding site (Figure 3B), the two fused rings engage in extensive π-π stacking and weak C-H/π interactions with aromatic residues such as Phe140 and Phe488, forming a hydrophobic binding core. The establishment of these aromatic protein–ligand stacking interactions appears to be a conserved feature among all known RSV fusion inhibitors and likely restricts the central heterocyclic moieties of these inhibitors to a fixed conformation [15].

**Figure 4:**
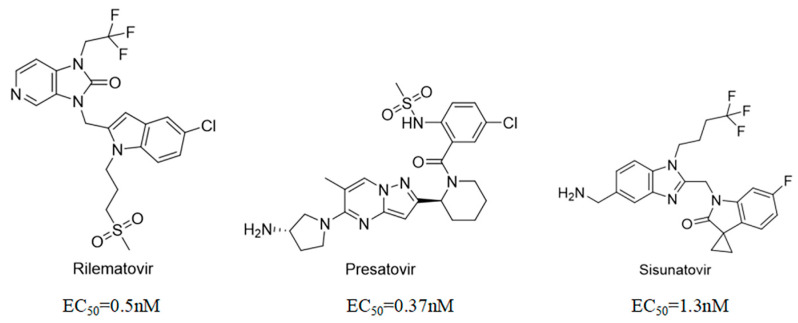
Structures of representative small-molecule inhibitors targeting F protein.

Well-defined protein structures, clearly characterized binding sites, and elucidated mechanisms of action have collectively accelerated rapid progress in related research. Multiple RSV F inhibitors have now advanced into the clinical pipeline, for example, Rilematovir, Sismatovir (RV521) (mean IC_50_ = 1.3 nM vs. RSV-A/B strains in Balb/c mice) [18], and Presatovir (GS-5806) (mean EC_50_ = 0.37 nM, range 0.15–1.09 nM, vs. rgRSV-A/HEp-2) [19] (Figure 4). However, currently reported small-molecule inhibitors targeting the F protein exhibit limited structural diversity. Multiple reports have been published to elucidate the drug resistance of this class of compounds [20,21]. Research has revealed that both K394R and K394H mutations in the F protein confer cross-resistance to RSV fusion inhibitors. For example, the single K394R mutation in the F protein confers 1250-fold resistance to BMS-433771, while it confers 6024-fold increased resistance to Rilematovir [22].

Marine natural products (MNPs) represent a rich source of structurally novel bioactive compounds [23]. Marine natural products have proven to be effective biological modulators, with several marine-derived compounds already approved for clinical use [24]. Their unique structures and bioactivities continue to drive interest in their application for drug discovery.

Computer-aided drug design (CADD) is an emerging field that has drawn considerable interest because of its potential to expedite and lower the cost of the drug development process [25]. Drug discovery is an expensive and time-consuming process, frequently taking 10–15 years for a drug to reach the market. Given these challenges, CADD has substantially changed research in the field by reducing the time and cost associated with drug development [26].

In this study, we applied a CADD strategy to systematically screen the Comprehensive Marine Natural Products Database (CMNPD, https://cmnpd.org/) [27] for novel F-protein inhibitors [28]. The process involved molecular docking, ADMET filtering, and MM/GBSA binding free energy calculations to identify promising, drug-like candidates. Through integration of all results, compounds exhibiting potential anti-RSV activity and optimal drug-like properties were identified.

## 2. Results

**Figure 5:**
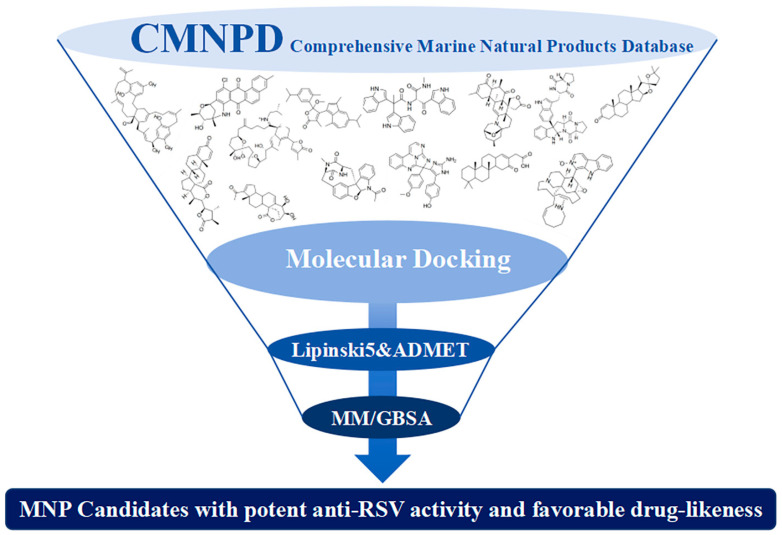
Schematic diagram of virtual screening workflow. Example marine-derived natural products from the CMNPD database are shown for visual representation.

All 31,561 molecules in the CMNPD were initially screened for their target binding poses and scores using molecular docking (Figure 5). The top ranked compounds were subsequently screened using integrated absorption, distribution, metabolism, excretion, and toxicity (ADMET) predictions [29]. Considering the inherent limitations of molecular docking, which operates on highly simplified computational models and performs basic calculations primarily based on shape complementarity between ligands and the protein binding pocket, only a narrow range of interactions were considered in the scoring function. Consequently, MM/GBSA was employed in conjunction with molecular docking to refine the estimation of binding energies. A significant strength of this approach lies in its capacity to treat both the ligand and the protein with flexibility, thereby accommodating the structural adaptations necessary for induced fit binding. MM/GBSA additionally incorporated more comprehensive energy terms, such as gas-phase molecular mechanical energy, solvation free energy, and entropy changes. The calculated total MM/GBSA binding free energy was decomposed into per-residue contributions. MM/GBSA calculations were additionally performed to enable a more in-depth analysis of their binding modes [30,31].

### 2.1. Molecular Docking

Molecular docking was conducted using AutoDock Vina to evaluate the anti-RSV potential of MNPs targeting the F protein (chains A, B, C). The co-crystal structure of the F protein with Rilematovir (PDB code: 5KWW) is depicted in Figure 3 [15]. All 31,561 MNPs (47,450 configurations) in the CMNPD were docked. The conformation with the lowest binding energy for each ligand was regarded as the most favorable conformation. The results for compounds with binding energies below −12.0 kcal/mol are compiled in Table S1. The co-crystallized Rilematovir and Sisunatovir, two known RSV F inhibitors, were used as positive controls.

**Figure 6:**
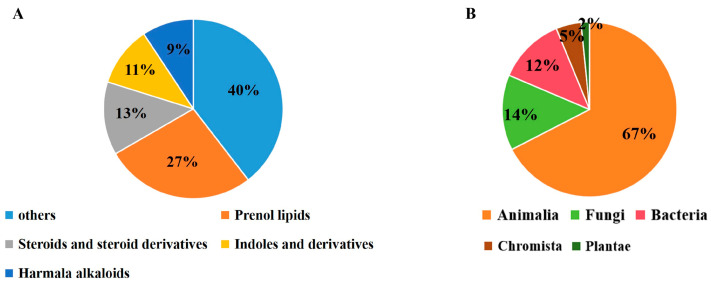
Distribution of 129 identified compounds. (**A**) Structural classes. (**B**) Source organisms.

A total of 129 compounds exhibited binding energy ranging from −15.9 to −12 kcal/mol, all lower than those of Rilematovir (−8.2 kcal/mol) and Sisunatovir (−10.5 kcal/mol). Chemically, the compounds primarily belong to indoles and derivatives (14), harmala alkaloids (12), prenol lipids (35), and steroids and steroid derivatives (17) (Figure 6A), sharing an extended rigid hydrophobic core strategically incorporated with heteroatoms. These compounds were derived from diverse biological kingdoms. The majority were from Animalia (87), followed by Fungi (18), Bacteria (16), Chromista (6), and Plantae (2) (Figure 6B). Most compounds were obtained from marine invertebrates, specifically sponges and corals. Fungal-origin compounds were isolated from Aspergillus, Penicillium, and Guignardia genera. Bacterial compounds were nearly all from Streptomyces species, classic producers of antibiotics and halogenated compounds.

### 2.2. Lipinski’s Rule of Five and ADMET Profile

The 129 compounds were evaluated for drug-likeness using Lipinski’s rules and ADMET prediction. Their properties, including rule compliance and ADMET predictions are summarized in Table S2.

Preliminary filtering applied Lipinski’s Rules: molecular weight ≤ 500 Da; calculated octanol–water partition coefficient (LogP) ≤ 5; hydrogen bond acceptors ≤ 10; hydrogen bond donors ≤ 5; estimated oral absorption ≥ 30% [29]. The final criterion was not strictly applied due to the inherent challenges in accurately predicting oral absorption *in silico*. It was found that 18 out of the 129 compounds violated more than two rules and were thereby excluded from further consideration.

**Figure 7:**
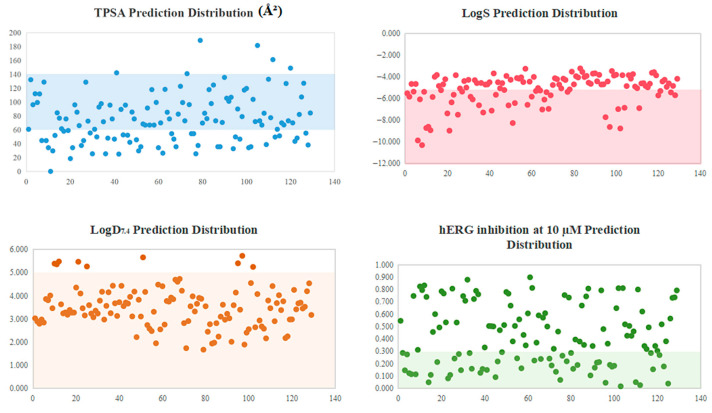
Distribution profiles of predicted properties for 129 compounds. Compounds are ordered by binding energy (1 = strongest). Translucent bands mark acceptable ranges: logS ≥ −5 (green), logD_7.4_ < 5 (orange), TPSA 60–140 Å^2^ (blue), and hERG < 0.3 (yellow).

ADMET properties were predicted using ADMETlab 3.0 [32]. Drug-likeness is largely determined by key properties such as aqueous solubility, membrane permeability, and safety profiles. To capture these critical aspects, four parameters—logS, LogD_7.4_, TPSA, and hERG inhibition at 10 µM—were selected for compound screening (Figure 7). Compounds with logS < −5 have poor water solubility [33]. LogD_7.4_ builds on logP by incorporating physiological pH 7.4 [34]; TPSA 60–140Å^2^ correlates with adequate membrane permeability and oral absorption. hERG inhibition risk was assessed to prevent potential cardiotoxicity (drug-induced torsades de pointes) [35]. Integrating these parameters, compounds with superior drug-like properties were prioritized [36]. Of the 129 candidates, 106 (82.2%) complied with Lipinski’s Rule of Five (RO5). Among these, 71 (67.0%) met the LogS ≥ −5; 51 (71.8% of solubility-qualified) had appropriate TPSA (60–140Å^2^); and 31 (60.8% of TPSA-qualified) showed low hERG risk (inhibition < 30% at 10 µM). Finally, 31 compounds complying with Lipinski’s Rule and meeting the criteria (logS ≥ −5, logD_7.4_ < 5, TPSA 60–140 Å^2^, hERG inhibition < 0.3 at 10 µM) were selected (Table 1). The predicted ADMET properties for all 129 compounds are visualized in Figure 7.

**Figure 8:**
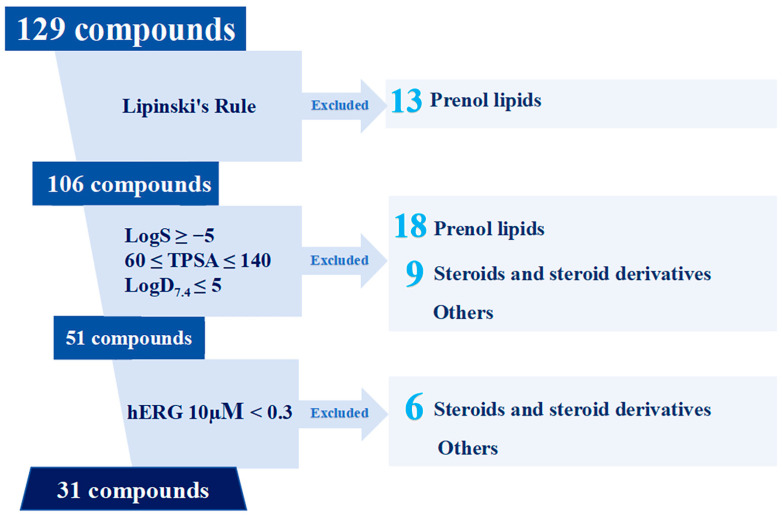
Dominant chemical classes in Lipinski properties and ADMET screening. The light blue numbers indicate the number of drug-like compounds in each category.

Based on ADMET predictions, 31 drug-like compounds were selected from the 129 compounds (Figure 8). Among these, indoles and derivatives (8) and harmala alkaloids (7) collectively accounted for half, remaining the most prevalent classes. The chemical classes of compounds excluded during the screening process based on Lipinski’s rule and ADMET criteria are shown in Figure 8.

Compound classes such as azepines, macrolactams, and naphthofurans exhibited minimal change in numbers after selection, indicating promising ADMET profiles. In contrast, only 1 of the 35 prenol lipids remained after screening, and only 2 of the 17 steroids remained. Detailed analysis revealed that the elimination of prenol lipids was primarily due to extremely poor aqueous solubility (high molecular weight and lipophilicity). A total of 13 molecules violated Lipinski’s Rule; of the remaining 22, 18 were excluded due to unfavorable TPSA and logS, and of the final 4, 3 were filtered out due to high hERG risk. Only CMNPD11217 met all criteria (Table 1). For steroids, high cardiotoxicity potential was the major cause of elimination; only 5 out of 17 had hERG < 0.3, while 3 of these 5 exhibited high lipophilicity, resulting in only 2 ultimately meeting all criteria.

The molecular weights of the 31 compounds range from 360 to 580 g/mol, with some exceeding the 500 g/mol benchmark for oral drugs. Since the positive control drug Rilematovir also exceeds this benchmark, we preliminarily considered these compounds within the common drug-like molecular weight range. Predicted logS values (−3.2 to −4.8) fall into the “soluble” to “moderately soluble” categories [37]. LogD_7.4_ values (1.7 to 3.7) indicate an optimal balance between hydrophilicity and lipophilicity, supporting both aqueous solubility and membrane permeability [38]. All TPSA values were below the 140 Å^2^ permeability threshold, with most under 100 Å^2^, suggesting good membrane permeability potential [39]. Predicted hERG inhibition probabilities are all <0.3, with over 60% (19 molecules) below 0.2, suggesting a favorable cardiac safety profile that mitigates cardiotoxicity risk in later development [36]. These compounds exhibit drug-likeness comparable to the positive controls.

### 2.3. Interaction Analysis

A detailed analysis of interactions between the 31 compounds and the F protein was conducted, identifying specific amino acid residues and interaction types (Table 2).

All 31 compounds interact with a critical set of conserved residues, with hydrophobic interactions being the dominant binding force. Hydrophobic interactions were observed for all compounds, primarily with Phe488, Phe140, and Phe137. Hydrogen bonding occurred in 22 compounds (~70%) involving residues such as Phe140, Gly139, Asp486, and Gln354. π-Stacking interactions were observed in 22 molecules, primarily with Phe140 and Phe488, stabilizing aromatic systems within the binding pocket. Notably, CMNPD31296 forms a salt bridge with Arg339C, indicating higher binding specificity and potential. Fourteen compounds engaged in all three interaction types (hydrophobic, hydrogen bonding, π-stacking) which may contribute to higher binding affinity and effective stabilization of the binding pocket. CMNPD25781 binds solely via hydrophobic interactions, underscoring the potency of hydrophobic interactions for this target.

### 2.4. MM/GBSA Binding Free Energy Calculation and Integrated Compound Assessment

The MM/GBSA approach was employed to calculate the binding free energy for the 31 molecules, serving as a rescoring parameter to optimize compounds identified from docking [31]. The results are summarized in Table 3, and the complete dataset is available in Table S3 in the Supplementary Materials.

The binding free energy (ΔGbind) was decomposed into molecular mechanics energy components to elucidate individual contributions. More negative values indicate stronger attractive forces; positive values represent unfavorable effects. Ligand Efficiency (LE) was also evaluated [40]. It is generally accepted that a ligand with |LE| ≥ 0.3 kcal·mol^−1^ per non-H atom indicates high efficiency and improved drug-likeness. All 31 compounds met this criterion, with |LE| ranging from 0.87 to 2.65, demonstrating effective target binding with minimalist structures. Total ΔGbind ranged from −111.15 to −34.04 kcal/mol, significantly different from the docking energy range (−15.9 to −12 kcal/mol), indicating that MM/GBSA provides a more reliable binding assessment.

Based on primary binding driving forces, compounds were categorized into three groups. Dominant electrostatic contribution (e.g., CMNPD6811, CMNPD29420, CMNPD15979): characterized by multiple polar or charged moieties that engage in electrostatic interactions but also create a desolvation penalty. Combined dominance of van der Waals forces and hydrophobic interactions (e.g., CMNPD6811, CMNPD29420, CMNPD15979): a classic binding mode with shape complementarity and hydrophobic driving force, often associated with enhanced drug-likeness. Unique binding mode (e.g., CMNPD11217, CMNPD21727, CMNPD25781): positive electrostatic interaction values, with CMNPD11217 showing favorable polar solvation energy in the bound state. Hydrogen bonding contributions were generally limited. In total, 11 compounds were identified with MM/GBSA binding free energies ranging from −111.15 to −57.57 kcal/mol and |LE| ≥ 2 kcal·mol^−1^ per non-H atom.

The 11 compounds meeting |LE| ≥ 2 include benzoxepines (1), carboxylic acids and derivatives (1), harmala alkaloids (7), macrolactams (1),, and naphthofurans (1). harmala alkaloids stand out in both quantity and for having the highest binding free energy, with the top seven compounds belonging to this class. CMNPD6811 (harmala alkaloids, ΔGbind = −111.15 kcal/mol) exhibits the most favorable binding energy. Other categories include CMNPD24939 (naphthofurans, −73.87 kcal/mol), CMNPD28415 (macrolactams, −70.22 kcal/mol), CMNPD19749 (carboxylic acid derivatives, −60.67 kcal/mol), and CMNPD10150 (benzoxepines, −57.57 kcal/mol).

Mechanistically, Coulomb energy, lipophilic energy, and van der Waals energy are primary drivers for harmala alkaloids, with significantly negative ΔECoulomb values. Despite a large desolvation penalty (positiveΔGGB), the net effect remains highly favorable. Structurally, harmala alkaloids feature bulky, rigid polycyclic skeletons that engage in extensive hydrophobic interactions and π-π stacking with hydrophobic/aromatic residues (primarily phenylalanine, Phe), consistent with the findings presented in Table 4. For the other four compounds, binding is primarily driven by ΔEvdW and ΔGLipo. Hydrogen (ΔEHbond) contributions are limited for all 11 compounds, aligning with the docking results.

**Figure 9:**
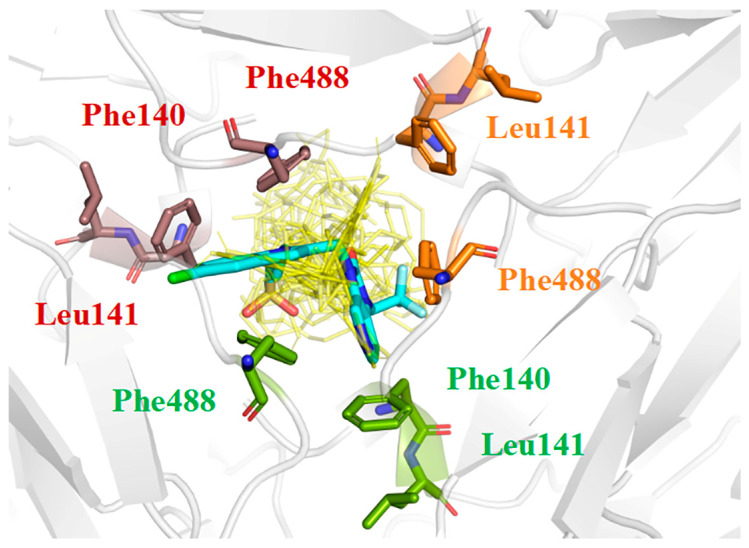
Superimposition of the Rilematovir-RSV-F co-crystal structure (PDB code: 5KWW) and docked poses of the 11 MNPs. MNPs are shown as thin yellow lines, and Rilematovir as a thick blue line, with atom-specific coloring (C: light blue, N: dark blue, O: red, S: orange). The three protein monomers are colored distinctly (orange, green, deep pink).

All 11 MNPs bind to the same active site as the positive control drug Rilematovir (Figure 9). The top six manzamine alkaloids exhibit highly consistent binding modes: the main driving force is electrostatic interactions (ΔECoulomb −120.62 to −137.26 kcal/mol); the primary antagonistic component is desolvation penalty (ΔGGB 106.20 to 138.69 kcal/mol); binding stability relies on hydrophobic interactions (ΔGLipo −40.58 to −49.61 kcal/mol) and van der Waals forces (ΔEvdW −46.70 to −64.29 kcal/mol); and hydrogen bonding (ΔEH bond) contribution is negligible. These results demonstrate that manzamine alkaloids achieve potent binding to the target pocket primarily through dominant electrostatic interactions, albeit with a substantial desolvation penalty, while their bulky, rigid scaffolds enable tight integration via hydrophobic and van der Waals contacts. In contrast, manzamine B N-oxide exhibits markedly reduced ΔECoulomb (−19.2 kcal/mol), likely due to N-oxide modification introducing steric hindrance and reducing effective positive charge, diminishing electrostatic attraction. Its ΔGGB is reduced to 57.92 kcal/mol, suggesting altered polarity and reduced desolvation penalty, though insufficient to compensate for lost electrostatic attraction. ΔGLipo and ΔEvdW remain largely unchanged, indicating that the scaffold still accesses the binding pocket, but crucial anchoring may be partially lost.

### 2.5. Binding Mode Interactions

**Figure 10:**
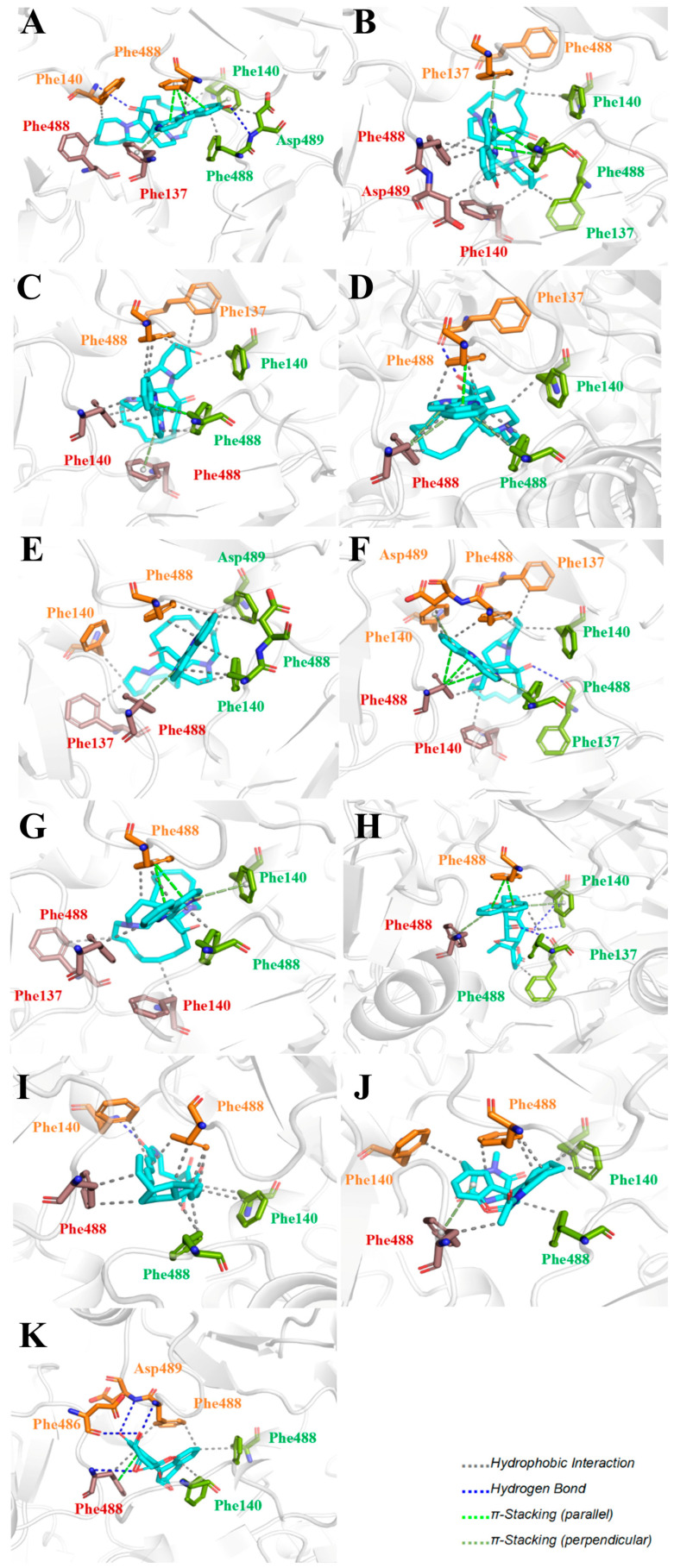
Three-dimensional representation of molecular docking models for 11 MNPs with the RSV-F trimer (PDB code: 5KWW). (**A**) CMNPD6811 (6-hydroxymanzamine A|manzamine Y); (**B**) CMNPD11950 (manzamine F); (**C**) CMNPD3271 (manzamine E); (**D**) CMNPD29420 (11-hydroxymanzamine J); (**E**) CMNPD15979 (8-hydroxymanzamine B); (**F**) CMNPD9689 (manzamine M); (**G**) CMNPD29418 (manzamine B N-oxide); (**H**) CMNPD24939 (rhytidenone A); (**I**) CMNPD28415 (pactamide E); (**J**) CMNPD19749 (azonazine); and (**K**) CMNPD10150 (spiroxin D). Ligands are shown with atom-specific coloring (C: light blue, N: dark blue, O: red). The three protein monomers are depicted in distinct colors (orange, green, deep pink). Non-covalent interactions are represented by dashed lines: hydrophobic (grey), hydrogen bonds (dark blue), parallel π-stacking (light green), and perpendicular π-stacking (grey-green).

Specific interactions between the 11 compounds and the F protein were explored (Figure 10). These compounds feature conjugated systems engaging in extensive hydrophobic interactions and π-stacking with the phenyl ring of Phe residues in the F protein. Additionally, heteroatoms can form hydrogen bonds with adjacent amino acid residues, such as Phe and Asp.

The detailed bond length data are presented in Table S4 in the Supplementary Materials. The hydrophobic interaction distances range from 2.0 to 4.0 Å, indicating effective hydrophobic contacts between the amino acid residues of the target protein and the ligand, which further confirms that hydrophobic interactions are a key driving force in small molecule–protein binding. The H–A distances of the hydrogen bonds are mostly in the range of 2.3–3.0 Å, indicating weak to moderate interactions and suggesting that hydrogen bonding plays a relatively minor role. Most π-stacking distances exceed 4.0 Å, indicating weak interactions with limited contribution to binding.

These compounds contain macrocyclic or complex polycyclic skeletons with multiple hydroxyl, carbonyl, or nitrogen substituents. The polycyclic skeletons of manzamine alkaloids generate significant hydrophobic, π-stacking, and van der Waals interactions, driving protein binding and conferring molecular rigidity. Flexibility from alkyl chains and rotatable bonds allows for conformational adaptation for optimal complementarity, enhancing drug-likeness. Rhytidenone A, azonazine, and spiroxin D represent another type: highly rigid and structurally compact. Rhytidenone A is planar and likely binds to the F protein via planar stacking; azonazine (bicyclo[3.3.1]nonane core) and spiroxin D (helical structure) possess fixed 3D shapes suggesting lock-and-key binding. These compounds have lower molecular weights than manzamine alkaloids. The nitrogen and oxygen atoms embedded in rigid, non-polar scaffolds contribute to moderate aqueous solubility. All these factors collectively contribute to optimal LogP and TPSA, thereby conferring favorable drug-like characteristics. Pactamide E, a macrolactam, exhibits intermediate flexibility and molecular weight, with a hydrophobic skeleton (alkyl chain) and hydrophilic functional groups (amide, hydroxyl) jointly contributing to favorable properties.

## 3. Discussion

In our study, potential anti-RSV molecules were screened using CADD with the CMNPD. Based on molecular docking, ADMET profiling, and MM/GBSA calculations, 11 compounds were identified as promising anti-RSV inhibitors targeting the F protein, including manzamine alkaloids (6-hydroxymanzamine A/manzamine Y, manzamine F, manzamine E, 11-hydroxymanzamine J, 8-hydroxymanzamine B, manzamine M, manzamine B N-oxide), the naphthofuran rhytidenone A, the macrolactam pactamide E, the carboxylic acid derivative azonazine, and the benzoxepine spiroxin D. All 11 compounds primarily interact with the RSV-F protein through Coulomb energy, lipophilic energy, and van der Waals energy. Molecular docking revealed superior binding affinity compared to the established F protein inhibitors Rilematovir and Sisunatovir. These compounds exhibit favorable predicted ADMET properties, indicating a high likelihood of good oral absorption and promising drug-like characteristics. The MM/GBSA-calculated binding free energies for all these compounds were highly favorable, ranging from −111.15 to −57.57 kcal/mol, indicating their predicted strong binding affinity to the target protein. However, it should be noted that the MM/GBSA approach involves several approximations (e.g., implicit solvent, neglect of entropy), which may overestimate absolute binding free energies. Thus, these calculated values are interpreted as relative ranking scores rather than absolute thermodynamic quantities [31].

Manzamine alkaloids, a unique group of β-carboline alkaloids isolated from marine sponges with broad bioactivities, such as cytotoxic, antibacterial, antifungal, antimalarial, insecticidal, anti-inflammatory, and anti-HIV activities [41], stood out due to exceptionally high binding energy and great drug-like properties. Yousaf et al. (2004) reported anti-HIV-1 activity for multiple manzamines (EC_50_ = 0.59–22.2 µM) and the first pharmacokinetic study of manzamine A, revealing absolute oral bioavailability of 20.6%, C_max_ = 1066 ng/mL, and T_max_ = 10 h, indicating good absorption and moderate oral bioavailability [42]. Palem et al. (2011) reported that manzamine A inhibited HSV-1 replication in SIRC cells at 1 µM, comparable to 50 µM acyclovir [43]. These data demonstrated broad-spectrum antiviral activity consistent with our virtual screening results.

In summary, 11 compounds, especially Manzamine alkaloids, are proposed as orally active anti-RSV drug candidates with RSV F inhibitory potential. Validating these *in silico* findings through in vitro and in vivo studies will be crucial for advancing these ligands as promising anti-RSV drug candidates targeting the F protein, or as starting points for further lead optimization.

However, this study has certain limitations. The current work primarily relied on conventional molecular docking as the main screening tool. While efficient for large-scale virtual screening, this static approach does not fully capture the dynamic behavior and electronic properties of the alkaloid–F protein interactions. Therefore, future studies could employ molecular dynamics simulations to assess binding stability and conformational flexibility over time, providing a more dynamic understanding of the inhibition mechanisms [44]. Additionally, PPI network analysis could be introduced to explore interactions between RSV-F protein and host factors [45], offering a system-level perspective on viral infection. Furthermore, DFT-based methods could be applied in two complementary directions: first, to validate the structural assignments of the screened alkaloids via NMR calculations [46]; and second, to investigate their electronic properties, global reactivity descriptors (such as hardness/softness), and local reactive sites (via Fukui indices) at the atomic level, thereby elucidating the intrinsic chemical reactivity that may underlie their binding affinities [47]. While these computational results provide a promising starting point for drug discovery, they need to be corroborated by subsequent in vitro experimental studies to assess their actual biological activity.

## 4. Materials and Methods

### 4.1. Docking Procedures

The chemical structures of 31,561 MNPs were obtained from the CMNPD (State Key Laboratory of Natural and Biomimetic Drugs, School of Pharmaceutical Sciences, Peking University, Beijing, China). Two clinically studied F protein inhibitors, Rilematovir and Sisunatovir, were used as positive controls; their 3D structures were retrieved from the PubChem database (National Center for Biotechnology Information, U.S. National Library of Medicine, Bethesda, MD, USA). The F protein (PDB code: 5KWW) was downloaded from the Protein Data Bank (PDB) database (Rutgers, The State University of New Jersey, Piscataway, NJ, USA). Molecular docking was performed using the AutoDock 4.2.6 software package (Molecular Graphics Laboratory, The Scripps Research Institute, La Jolla, CA, USA), according to the standard procedure for a rigid receptor and a flexible ligand. Initial screening parameters were as follows: num_modes = 5 and exhaustiveness = 10. Subsequent docking used num_modes = 20 and exhaustiveness = 20 for accuracy. The grid size was 25.0 Å in x, y, and z directions, centered on co-crystallized ligand coordinates (18.1, 19.6, 21.1). The specified coordinates were used during the docking of each ligand. The two F protein inhibitors, Rilematovir and Sisunatovir, were used as positive controls. AutoDockTools (ADT) v.1.5.6 (Molecular Graphics Laboratory, The Scripps Research Institute, La Jolla, CA, USA) and PyMOL Molecular Graphics System v.3.1.6.1 (Schrödinger, LLC, New York, NY, USA) were used for preparation and visualization.

### 4.2. Prediction of ADMET Profile

ADMET properties (e.g., absorption, distribution, metabolism, excretion, and toxicity) were predicted using the ADMETlab 3.0 online platform [32], employing a comprehensive collection of predictive models, including machine learning algorithms and quantitative structure–activity relationship (QSAR) approaches, trained on extensively curated chemical datasets. For key parameters, the standard pre-set prediction thresholds of the platform were applied to evaluate drug-likeness and medicinal chemistry compatibility. All predictions were conducted using the default parameters as implemented in the webserver.

### 4.3. Binding Free Energy Calculations

Binding free energies were calculated using molecular mechanics/generalized born surface area (MM/GBSA) in Schrödinger Suite (Prime module, Maestro v.13.5.128, Schrödinger, LLC, New York, NY, USA, 2023). The calculations were performed using the OPLS4 force field and VSGB solvation model to represent the aqueous environment. All other parameters were maintained at their default settings [48].

### 4.4. Two-Dimensional Representation

Two-dimensional (2D) protein–ligand interaction diagrams were generated using the Ligand Interactions module of Molecular Operating Environment (MOE), v.2024.06 (Chemical Computing Group ULC, Montreal, QC, Canada).

## Figures and Tables

**Table 1:** Lipinski properties (0: adherence; 1: violation), LogS, LogD_7.4_, TPSA (Å^2^), and hERG (10 µM) for the 31 selected ligands.

CMNPD ID	MW (g/mol)	Lipinski’s Rules	LogS	LogD_7.4_	TPSA (Å^2^)	hERG (10 µM)
CMNPD29188	510.634	0	−4.676	2.794	96.1	0.146
CMNPD27687	510.634	0	−4.676	2.844	99.26	0.123
CMNPD29420	568.806	0	−4.019	3.240	84.41	0.050
CMNPD15979	566.79	0	−3.847	3.280	76.71	0.110
CMNPD6811	564.774	0	−4.718	3.271	75.62	0.213
CMNPD9692	584.849	0	−3.866	3.152	65.97	0.109
CMNPD3271	564.774	0	−4.410	3.072	72.46	0.278
CMNPD11950	580.773	0	−4.324	2.974	92.69	0.285
CMNPD18887	649.913	0	−4.596	3.564	97.58	0.159
CMNPD28415	476.617	0	−4.731	3.675	95.5	0.126
CMNPD9689	564.774	0	−4.718	3.129	75.62	0.157
CMNPD13935	384.395	0	−3.935	2.215	85.51	0.293
CMNPD24939	448.471	0	−4.061	2.476	91.29	0.243
CMNPD18676	564.646	0	−3.273	1.950	117.85	0.163
CMNPD26179	517.589	0	−4.776	2.820	122.64	0.241
CMNPD31296	539.669	0	−4.148	1.738	99.21	0.186
CMNPD21727	452.591	0	−4.714	3.547	69.67	0.287
CMNPD23553	447.535	0	−3.962	2.448	83.8	0.158
CMNPD27283	564.646	0	−3.240	1.937	117.85	0.189
CMNPD29418	566.79	0	−3.724	2.967	70.53	0.105
CMNPD1440	520.707	0	−4.429	3.029	105.45	0.168
CMNPD10150	364.309	0	−3.836	2.015	101.05	0.209
CMNPD11737	692.958	0	−4.726	3.592	106.81	0.213
CMNPD16065	460.611	0	−4.446	3.399	89.9	0.046
CMNPD19749	403.438	0	−3.480	1.894	78.95	0.190
CMNPD19768	479.533	0	−3.899	2.410	117.2	0.176
CMNPD22201	564.646	0	−3.823	2.557	119.54	0.183
CMNPD25781	506.643	0	−3.865	2.641	103.78	0.017
CMNPD17925	503.711	0	−3.648	2.175	67.01	0.286
CMNPD21065	538.608	0	−3.587	2.249	126.64	0.155
CMNPD11217	608.86	0	−4.624	3.452	107.22	0.039
Rilematovir ^a^	500.09	0	−4.079	2.501	78.89	0.445
Sisunatovir ^a^	446.17	0	−3.338	2.599	64.15	0.769

^a^ Rilematovir and Sisunatovir are known RSV fusion (F) protein inhibitors and were used as positive controls.

**Table 2:** Interacting amino acid residues between the 31 compounds and the F Protein. A, B, and C denote distinct monomers of the F protein.

CMNPD ID	Structure	Hydrogen Bonds	Hydrophobic Interactions	π-Stacking	Salt Bridges
CMNPD29188	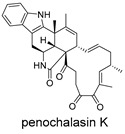		Phe140C, Phe488A, Phe488B, Phe488C, Asp489A	Phe140A, Phe488B	
CMNPD27687	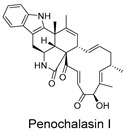		Phe140C, Phe488A, Phe488B, Phe488C, Asp489B	Phe140B, Phe488C	
CMNPD29420	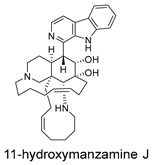	Phe137C	Phe140B, Phe488A, Phe488B, Phe488C	Phe488A, Phe488B, Phe488C	
CMNPD15979	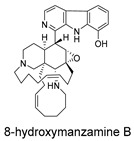		Phe137A, Phe140B, Phe140C, Phe488B, Phe488C, Asp489B	Phe488A	
CMNPD6811	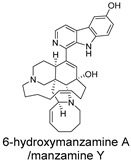	Phe140C, Asp489B	Phe137A, Phe140B, Phe140C, Phe488B, Phe488C, Asp489B	Phe140B, Phe488A, Phe488C	
CMNPD9692	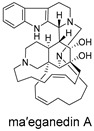		Phe137A, Phe140A, Phe140C, Phe488A, Phe488B, Phe488C	Phe488A	
CMNPD3271	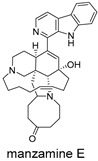		Phe137C, Phe140B, Phe488A, Phe488B, Phe488C	Phe140A, Phe488B	
CMNPD11950	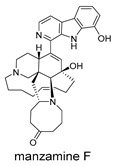		Phe137B, Phe137C, Phe140A, Phe140B, Phe488A, Phe488B, Phe489A	Phe488B, Phe488C	
CMNPD18887	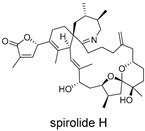	Phe140C	Phe137A, Phe137C, Phe140B, Val144C, Gln354A, Phe488A, Phe488C		
CMNPD28415	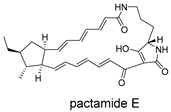	Phe140C	Phe140B, Phe140C, Phe488A, Phe488B, Phe488C		
CMNPD9689	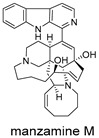	Phe137B	Phe137C, Phe140A, Phe140B, Phe488A, Phe488C, Phe489C	Phe140C, Phe488A, Phe488B	
CMNPD13935	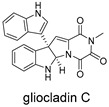	Asp486A	Phe488A, Phe488B, Phe488C, Phe489B	Phe140A, Phe140B, Phe488A, Phe488B, Phe488C	
CMNPD24939	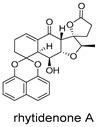	Phe137B, Phe139B, Phe140B	Phe137B, Phe140B, Phe488B	Phe140B, Phe488A, Phe488C	
CMNPD18676	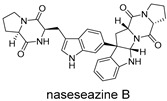	Gly139B, Phe140B, Asp486A	Phe137C, Phe140B, Phe140C, Phe488A, Phe488B, Phe488C, Asp489C	Phe488C	
CMNPD26179	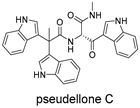	Phe137B	Phe137B, Phe140A, Phe488A, Phe488B, Phe488C, Asp489A, Asp489B	Phe140A, Phe140B, Phe488B, Phe488C	
CMNPD31296	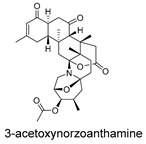	Phe137C	Phe140B, Phe140C, Phe488A, Phe488B, Phe488C		Arg339C
CMNPD21727	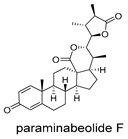	Gln354C	Phe137A, Phe140A, Phe488A, Phe488B, Phe488C	Phe488A	
CMNPD23553	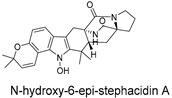	Gly139A, Phe140A	Phe137A, Phe140C, Phe488A, Phe488B, Phe488C	Phe488A, Phe488B	
CMNPD27283	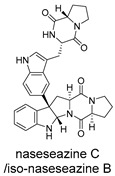	Phe137C, Gly139C	Phe140B, Gln354C, Phe488A, Phe488B, Phe488C	Phe140C, Phe488A, Phe488C	
CMNPD29418	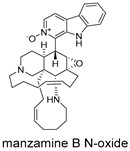		Phe137A, Phe140A, Phe488A, Phe488B, Phe488C	Phe140B, Phe488C	
CMNPD1440	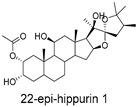	Phe140B, Phe488C, Asp489C	Phe140B, Gln354B, Phe488A, Phe488B, Phe488C		
CMNPD10150	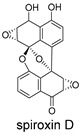	Asp486A, Phe488A, Phe488C, Asp489C	Phe140B, Phe488A, Phe488B, Phe488C	Phe488A	
CMNPD11737	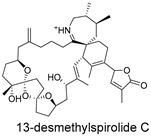	Gln354A	Leu142C, Val144C, Gln354A, Phe488B	Phe488A	
CMNPD16065	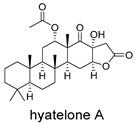	Gly139A, Phe140A, Gln354A	Phe137A, Phe488A, Phe488B, Phe488C		
CMNPD19749	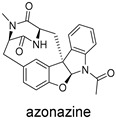		Phe140B, Phe140C, Phe488A, Phe488B, Phe488C	Phe488A	
CMNPD19768	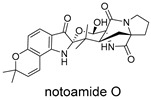	Phe137B, Phe140B	Phe140C, Phe488A, Phe488B, Phe488C	Phe488C	
CMNPD22201	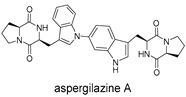	Asp486A, Lys498A	Phe488A, Phe488B, Asp489B	Phe140B, Phe488C	
CMNPD25781	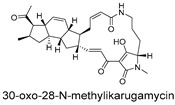		Phe488A, Phe488B, Phe488C		
CMNPD17925	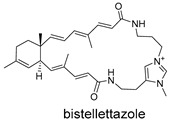	Phe137B	Phe488A, Phe488B, Phe488C		
CMNPD21065	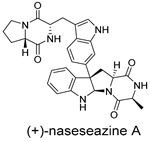	Phe137A, Arg339A	Phe140B, Phe140C, Phe488A		
CMNPD11217	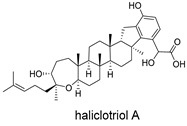	Phe488C	Phe137A, Phe140C, Gln354A, Phe488B, Phe488C		

**Table 3:** MM/GBSA binding free energies determined for the selected MNPs. Energy terms: binding free energy (ΔGbind); Coulomb energy (ΔECoulomb); hydrogen bonding energy (ΔEHbond); lipophilic energy (ΔGLipo); Van der Waals energy (ΔEvdW); generalized Born solvation energy (ΔGGB) in kcal/mol; Ligand Efficiency (LE) in kcal·mol^−1^ per non-H atom.

CMNPD ID	Molecular Docking	ΔGbind	ΔECoulomb	ΔEHbond	ΔGLipo	ΔGGB	ΔEvdW	LE
CMNPD6811	−13.3	−111.15	−136.28	−0.03	−41.54	125.08	−50.78	−2.65
CMNPD11950	−12.9	−109.15	−120.62	−0.05	−43.29	106.20	−46.70	−2.54
CMNPD3271	−13.1	−108.29	−119.98	0.17	−46.28	122.47	−59.13	−2.58
CMNPD29420	−13.4	−106.94	−132.50	0.17	−42.45	136.90	−59.93	−2.55
CMNPD15979	−13.4	−104.85	−137.26	0.08	−49.61	138.69	−55.94	−2.50
CMNPD9689	−12.8	−102.39	−128.75	−0.44	−40.58	131.14	−65.21	−2.44
CMNPD29418	−12.2	−84.44	−19.20	1.24	−47.39	57.92	−64.29	−2.01
CMNPD27283	−12.3	−80.77	−52.41	0.76	−36.44	69.97	−66.45	−1.92
CMNPD9692	−13.1	−79.37	−77.58	0.34	−43.69	99.78	−60.42	−1.85
CMNPD11737	−12.2	−75.95	−4.47	−0.89	−40.20	24.49	−65.62	−1.52
CMNPD18887	−12.9	−73.90	−36.81	−0.19	−40.43	51.83	−57.33	−1.57
CMNPD24939	−12.5	−73.87	−46.59	−0.34	−29.88	56.07	−44.81	−2.24
CMNPD29188	−14.7	−71.74	−39.00	−1.53	−31.40	57.45	−54.67	−1.89
CMNPD28415	−12.8	−70.22	−27.82	−0.22	−38.12	51.82	−56.41	−2.01
CMNPD22201	−12.2	−66.28	−17.18	−0.20	−27.28	38.18	−54.04	−1.58
CMNPD18676	−12.5	−64.48	−41.78	−0.46	−35.45	69.81	−59.46	−1.54
CMNPD31296	−12.4	−63.59	−30.76	0.19	−32.17	53.99	−55.72	−1.63
CMNPD19749	−12.2	−60.67	−32.21	0.84	−41.15	65.78	−52.20	−2.02
CMNPD21727	−12.3	−58.85	6.85	0.24	−35.68	15.24	−46.32	−1.78
CMNPD27687	−14.5	−57.64	−35.98	0.98	−36.76	71.92	−57.49	−1.52
CMNPD13935	−12.6	−57.63	−22.97	1.24	−25.17	47.31	−50.19	−1.99
CMNPD10150	−12.2	−57.57	−59.61	−0.15	−35.53	86.89	−46.16	−2.13
CMNPD23553	−12.3	−56.51	−34.95	−0.32	−27.23	68.31	−56.38	−1.71
CMNPD16065	−12.2	−55.89	−35.28	−0.43	−30.49	42.40	−40.63	−1.69
CMNPD1440	−12.2	−55.57	−42.71	0.54	−29.44	60.23	−43.99	−1.50
Rilematovir ^a^	−8.2	−33.38	−7.34	−0.24	−23.06	56.53	−53.65	−1.01
Sisunatovir ^a^	−10.5	−75.80	−113.05	−0.86	−36.22	131.60	−47.74	−2.36

^a^ Rilematovir and Sisunatovir are known RSV fusion (F) protein inhibitors and were used as positive controls.

**Table 4:** Structures of final compounds identified through screening.

CMNPD ID	Name	Structure	Class	ΔGbind (kcal/mol)
CMNPD6811	6-hydroxymanzamine A|manzamine Y	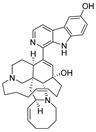	Harmala alkaloids	−111.15
CMNPD11950	manzamine F	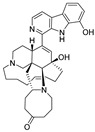	Harmala alkaloids	−109.15
CMNPD3271	manzamine E	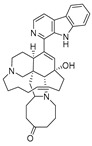	Harmala alkaloids	−108.29
CMNPD29420	11-hydroxymanzamine J	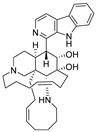	Harmala alkaloids	−106.94
CMNPD15979	8-hydroxymanzamine B	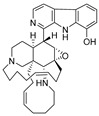	Harmala alkaloids	−104.85
CMNPD9689	manzamine M	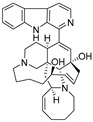	Harmala alkaloids	−102.39
CMNPD29418	manzamine B N-oxide	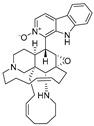	Harmala alkaloids	−84.44
CMNPD24939	rhytidenone A	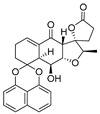	Naphthofurans	−73.87
CMNPD28415	pactamide E	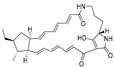	Macrolactams	−70.22
CMNPD19749	azonazine	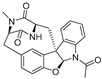	Carboxylic acids and derivatives	−60.67
CMNPD10150	spiroxin D	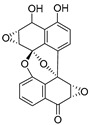	Benzoxepines	−57.57

## Data Availability

The original data supporting this study (CMNPD database) are available from the corresponding author upon reasonable request. The CMNPD database is publicly accessible at [http://www.cmnpd.org/]. The newly generated data (e.g., molecular docking results, ADMET predictions, and MM/GBSA calculation data of the screened compounds) are available in the Supplementary Materials accompanying this article.

## Supplementary Materials

The following supporting information can be downloaded at: https://www.mdpi.com/article/10.3390/ijms27052484/s1.
