# Supplementary material for: Virtual Screening of Marine Natural Products Targeting the F Protein for Anti-RSV Drug Discovery
Source: Int J Mol Sci. 2026 Mar 8;27(5):2484. doi: 10.3390/ijms27052484 (PMC12985475; doi:10.3390/ijms27052484)
Supplement: Supplementary file 1 [file ijms-27-02484-s001.zip › ijms-4167446-supplementary.pdf]

Supplementary Materials

Virtual Screening of Marine Natural Products
Targeting the F Protein for Anti-RSV Drug Discovery

Wenqing Liu et al.

Table S1. Molecular docking results for compounds with binding energies below -12.0 kcal/mol.

CMNPD ID	ΔGb (kcal/mol)	Name	Class	Kingdom-Species
CMNPD6532	-15.9	trisindoline	Indoles and derivatives	Bacteria-Vibrio sp.
CMNPD18841	-14.7	sporothrin B	Tetralins	Fungi-Sporothrix sp.
CMNPD29188	-14.7	penochalasin K	Indoles and derivatives	Fungi-Penicillium chrysogenum
CMNPD18840	-14.5	sporothrin A	Tetralins	Fungi-Sporothrix sp.
CMNPD27687	-14.5	Penochalasin I	Indoles and derivatives	Fungi-Penicillium chrysogenum
CMNPD27913	-14.4	petroquinone G	Anthracenes	Animalia-Petrosia (Petrosia) alfiani
CMNPD19652	-13.8	arsindoline A	Quinolines and derivatives	Bacteria-Aeromonas sp.
CMNPD27914	-13.8	petroquinone H	Anthracenes	Animalia-Petrosia (Petrosia) alfiani
CMNPD12678	-13.7	12,34-oxamanzamine A	Harmala alkaloids	Animalia-unidentified species of Family Petrosiidae
CMNPD22796	-13.7	anthogorgiene J	Prenol lipids	Animalia-Anthogorgia sp.
CMNPD8410	-13.6	2,2′-biguaiazulenyl	Prenol lipids	Animalia-Calicogorgia granulosa
CMNPD22800	-13.6	anthogorgiene N	Prenol lipids	Animalia-Anthogorgia sp.
CMNPD6529	-13.5	7,7-bis(3-indolyl)-p-cresol	Indoles and derivatives	Bacteria-Vibrio sp.
CMNPD29420	-13.4	11-hydroxymanzamine J	Harmala alkaloids	Animalia-Acanthostrongyloph ora sp.
CMNPD15979	-13.4	8-hydroxymanzamine B	Harmala alkaloids	Animalia-Acanthostrongyloph ora sp.
CMNPD12676	-13.4	ent-12,34-oxamanzamine E	Harmala alkaloids	Animalia-unidentified species of Family Petrosiidae
CMNPD19653	-13.4	arsindoline B	Indoles and derivatives	Bacteria-Aeromonas sp.
CMNPD6811	-13.3	6-hydroxymanzamine A | manzamine Y	Harmala alkaloids	Animalia-Amphimedon sp. Animalia-Haliclona sp.
CMNPD9231	-13.3	(22S,24S)-24-methyl-22,25-	Steroids and steroid	Animalia-Sarcophyton

		epoxyfurost-5-ene-3β,20b-diol	derivatives	crassocaule
CMNPD6287	-13.2	gorgiabisazulene	Prenol lipids	Animalia-Acalycigorgia sp.
CMNPD22799	-13.2	anthogorgiene M	Prenol lipids	Animalia-Anthogorgia sp.
CMNPD6204	-13.1	toxicol C	Prenol lipids	Animalia-Haliclona (Gellius) toxia
CMNPD7175	-13.1	longithorone A	Prenol lipids	Animalia-Aplidium longithorax
CMNPD9692	-13.1	ma′eganedin A	Indoles and derivatives	Animalia-Amphimedon sp.
CMNPD22798	-13.1	anthogorgiene L	Prenol lipids	Animalia-Anthogorgia sp.
CMNPD25128	-13.1	acanthomanzamine D	Harmala alkaloids	Animalia-Acanthostrongylophora ingens
CMNPD25797	-13.1	marangucycline B	Angucyclines	Bacteria-Streptomyces sp.
CMNPD3271	-13.1	manzamine E	Harmala alkaloids	Animalia-Xestospongia sp.
CMNPD5448	-13	manzamine A	Harmala alkaloids	Animalia-Ircinia sp.<br>Animalia-Amphimedon sp.<br>Animalia-Haliclona sp.
CMNPD17081	-13	cortistatin J	Isoquinolines and derivatives	Animalia-Corticium simplex
CMNPD187	-13	disidein	Prenol lipids	Animalia-Dysidea pallescens
CMNPD6203	-12.9	toxicol B	Prenol lipids	Animalia-Haliclona (Gellius) toxia
CMNPD11950	-12.9	manzamine F	Harmala alkaloids	Animalia-unidentified species of Family Petrosiidae
CMNPD18887	-12.9	spirolide H	Azepines	Chromista-Alexandrium ostenfeldii
CMNPD16617	-12.8	shearinine K	Naphthopyrans	Fungi-Penicillium sp.
CMNPD17082	-12.8	cortistatin K	Isoquinolines and derivatives	Animalia-Corticium simplex
CMNPD22676	-12.8	dimeric 6′-aueroxyaureol	Benzopyrans	Animalia-Smenospongia sp.
CMNPD28415	-12.8	pactamide E	Macrolactams	Bacteria-Streptomyces pactum
CMNPD9689	-12.8	manzamine M	Harmala alkaloids	Animalia-Amphimedon sp.
CMNPD5593	-12.7	luffarin F	Prenol lipids	Animalia-Luffariella geometrica
CMNPD6202	-12.7	toxicol A	Prenol lipids	Animalia-Haliclona (Gellius) toxia
CMNPD3766	-12.7	araguspongine B | araguspongine E	Piperidines	Animalia-Xestospongia sp.
CMNPD21931	-12.7	fradcarbazole A	Indoles and derivatives	Bacteria-Streptomyces fradiae
CMNPD24198	-12.7	sinubrasolide B	Steroids and steroid derivatives	Animalia-Sinularia brassica
CMNPD28413	-12.7	pactamide C	Macrolactams	Bacteria-Streptomyces pactum
CMNPD29417	-12.7	kepulauamine A	Harmala alkaloids	Animalia-Acanthostrongyloph

				ora sp.
CMNPD3767	-12.7	araguspongine C	Piperidines	Animalia-Xestospongia sp.
CMNPD13935	-12.6	gliocladin C	Indoles and derivatives	Fungi-Gliocladium sp.
CMNPD16561	-12.6	marmycin A	Angucyclines	Bacteria-Streptomyces sp.
CMNPD17083	-12.6	cortistatin L	Isoquinolines and derivatives	Animalia-Corticium simplex
CMNPD22801	-12.6	anthogorgiene O	Prenol lipids	Animalia-Anthogorgia sp.
CMNPD22876	-12.6	anthogorgiene Q	Prenol lipids	Animalia-Anthogorgia sp.
CMNPD24895	-12.6	guignardin F	Tetralins	Fungi-Guignardia sp.
CMNPD6258	-12.5	3,4-dihydroxypregna-5,17-diene-10,2-carbolactone	Steroids and steroid derivatives	Animalia-Strongylophora sp.
CMNPD24939	-12.5	rhytidenone A	Naphthofurans	Fungi-Rhytidhysteron sp.
CMNPD25312	-12.5	16-O-deacetyl-12,16-epi-scalarolbutanolide	Steroids and steroid derivatives	Animalia-Scalarispongia sp.
CMNPD18676	-12.5	naseseazine B	Indoles and derivatives	Bacteria-Streptomyces sp.
CMNPD31192	-12.5	dysiarenone	Benzopyrans	Animalia-Dysidea arenaria
CMNPD31416	-12.5	verrucorosteroid B	Prenol lipids	Animalia-Verrucella corona
CMNPD4409	-12.5	4-ene-3,6-diketosteroids	Steroids and steroid derivatives	Animalia-Geodia cydonium
CMNPD6288	-12.4	gorgiagallylazulene	Prenol lipids	Animalia-Acalycigorgia sp.
CMNPD7826	-12.4	No Data	Steroids and steroid derivatives	Animalia-Scleronephthya gracillimum
CMNPD10813	-12.4	longithorol A	Phenols 酚类	Animalia-Aplidium longithorax
CMNPD15432	-12.4	suberoretisteroid B	Prenol lipids	Animalia-Annella reticulata
CMNPD16562	-12.4	marmycin B	Angucyclines	Bacteria-Streptomyces sp.
CMNPD18523	-12.4	tagalsin L	Prenol lipids	Plantae-Ceriops tagal
CMNPD22793	-12.4	anthogorgiene G	Prenol lipids	Animalia-Anthogorgia sp.
CMNPD22794	-12.4	anthogorgiene H	Prenol lipids	Animalia-Anthogorgia sp.
CMNPD23584	-12.4	No Data	Naphthopyrans	Fungi-Penicillium camemberti
CMNPD26179	-12.4	pseudellone C	Indoles and derivatives	Fungi-Pseudallescheria ellipsoidea
CMNPD31296	-12.4	3-acetoxynorzoanthamine	Azaspirodecane derivatives	Animalia-Zoanthus pulchellus
CMNPD3986	-12.4	28-deoxyzoanthenamine	Azaspirodecane derivatives	Animalia-Zoanthus sp.
CMNPD3787	-12.3	jaspamide | jaspakinolide	Carboxylic acids and derivatives	Animalia-Jaspis sp.
CMNPD21352	-12.3	diplopuupehenone	Prenol lipids	Animalia-Dysidea sp.
CMNPD9104	-12.3	petrosaspongiolide K	Organooxygen compounds	Animalia-Petrosaspongia nigra
CMNPD14874	-12.3	flabellinone	Prenol lipids	Chromista-Stypopodium flabelliforme
CMNPD17079	-12.3	cortistatin G	Pyridines and derivatives	Animalia-Corticium simplex
CMNPD19255	-12.3	No Data	Steroids and steroid derivatives	Animalia-Axinella sp.

CMNPD21315	-12.3	hyrtioseragamine B	Quinolines and derivatives	Animalia-Hyrtios sp.
CMNPD21727	-12.3	paraminabeolide F	Steroids and steroid derivatives	Animalia-Paraminabea acronocephala
CMNPD23553	-12.3	N-hydroxy-6-epi-stephacidin A	Quinolines and derivatives	Fungi-Aspergillus taichungensis
CMNPD24892	-12.3	guignardin C	Naphthalenes	Fungi-Guignardia sp.
CMNPD27283	-12.3	naseseazine C | iso-naseseazine B	Indoles and derivatives	Bacteria-Streptomyces sp.
CMNPD27665	-12.3	preussomerin F	Tetralins	Fungi-Lasiodiplodia theobromae
CMNPD28333	-12.3	eudistidine C	Azoles	Animalia-Eudistoma sp.
CMNPD9167	-12.3	norzoanthamine	Azaspirodecane derivatives	Animalia-Zoanthus sp.
CMNPD4506	-12.3	No Data	Steroids and steroid derivatives	Animalia-Sinularia sp.
CMNPD7823	-12.2	No Data	Prenol lipids	Animalia-Scleronephthya gracillimum
CMNPD29418	-12.2	manzamine B N-oxide	Harmala alkaloids	Animalia-Acanthostrongylophora sp.
CMNPD8781	-12.2	asperazine	Indoles and derivatives	Fungi-Aspergillus niger
CMNPD1440	-12.2	22-epi-hippurin 1	Steroids and steroid derivatives	Animalia-Isis hippuris
CMNPD10150	-12.2	spiroxin D	Benzoxepines	Fungi-unidentified fungus
CMNPD11737	-12.2	13-desmethylspirolide C	Azepines	Chromista-Alexandrium ostenfeldii
CMNPD12052	-12.2	plakinamine F	Prenol lipids	Animalia-Corticium sp.
CMNPD12084	-12.2	(32R,33S,34S)-32,35-anhydrobacteriohopanetetrol	Prenol lipids	Animalia-Plakortis simplex
CMNPD16065	-12.2	hyatelone A	Naphthofurans	Animalia-Hyattella intestinalis
CMNPD16525	-12.2	No Data	Prenol lipids	Plantae-Barringtonia racemosa
CMNPD19749	-12.2	azonazine	Carboxylic acids and derivatives	Fungi-Aspergillus insulicola
CMNPD19768	-12.2	notoamide O	Benzopyrans	Fungi-Aspergillus sp.
CMNPD22201	-12.2	aspergilazine A	Carboxylic acids and derivatives	Fungi-Aspergillus taichungensis
CMNPD22791	-12.2	anthogorgiene E	Prenol lipids	Animalia-Anthogorgia sp.
CMNPD22797	-12.2	anthogorgiene K	Prenol lipids	Animalia-Anthogorgia sp.
CMNPD25781	-12.2	30-oxo-28-N-methylikarugamycin	Macrolactams	Bacteria-Streptomyces zhaozhouensis
CMNPD29424	-12.2	tulongicin	Indoles and derivatives	Animalia-Topsentia sp.
CMNPD30020	-12.2	urdamycin N5	Angucyclines	Bacteria-Streptomyces diastaticus
CMNPD3987	-12.2	22-epi-28-deoxyzoanthenamine	Azaspirodecane derivatives	Animalia-Zoanthus sp.

CMNPD6258	-12.1	3,4-dihydroxypregna-5,17-diene-10,2-carbolactone	Steroids and steroid derivatives	Animalia-Strongylophora sp.
CMNPD6260	-12.1	No Data	Steroids and steroid derivatives	Animalia-Strongylophora sp.
CMNPD8044	-12.1	cystoketal chromane	Benzopyrans	Chromista-Cystoseira amentacea
CMNPD8376	-12.1	polymastiamide C	Steroids and steroid derivatives	Animalia-Polymastia boletiformis
CMNPD1098	-12.1	No Data	Prenol lipids	Animalia-Hyrtios communis
CMNPD10398	-12.1	jaspamide C	Carboxylic acids and derivatives	Animalia-Jaspis splendens
CMNPD12051	-12.1	plakinamine E	Prenol lipids	Animalia-Corticium sp.
CMNPD6262	-12.1	biemnasterol	Steroids and steroid derivatives	Animalia-Biemna sp.
CMNPD14868	-12.1	atomarianone A	Prenol lipids	Chromista-Taonia atomaria
CMNPD1423	-12.1	(22R)-24ξ-methylcholest-5-ene-3β,22,25-triol	Steroids and steroid derivatives	Animalia- Lobophytum depressum
CMNPD17925	-12.1	bistellettazole	Azoles	Animalia-Stelletta sp.
CMNPD21065	-12.1	(+)-naseseazine A	Indoles and derivatives	Bacteria-Streptomyces sp.
CMNPD2092	-12.1	zoanthamine	Azaspirodecane derivatives	Animalia-Zoanthus sp.
CMNPD30029	-12.1	vineomycin D	Naphthacenes	Bacteria-Streptomyces sp.
CMNPD31420	-12.1	verrucorosteroid F	Steroids and steroid derivatives	Animalia-Verrucella corona
CMNPD5748	-12.1	minabeolide 3	Steroids and steroid derivatives	Animalia-Minabea sp.
CMNPD3954	-12.1	antheliolide B †	Lactones	Animalia-Anthelia glauca
CMNPD9792	-12	petrosaspongiolide M	Prenol lipids	Animalia-Petrosaspongia nigra
CMNPD11217	-12	haliclotriol A	Prenol lipids	Animalia-Haliclona sp.
CMNPD19934	-12	27-hydroxy-13-desmethyl spirolide C	Azepines	Chromista-Alexandrium ostenfeldii
CMNPD12818	-12	No Data	Prenol lipids	Animalia-Corticium sp.
CMNPD25333	-12	plakinamine N	Prenol lipids	Animalia-Corticium niger
CMNPD13777	-12	haouamine A	Indanes	Animalia-Aplidium haouarianum
Rilematovir [a]	-8.2			
Sisunatovir [a]	-10.5			

[a] Rilematovir and Sisunatovir are known RSV fusion (F) protein inhibitors and were used as positive controls.

Table S2. Summary of Lipinski's Rules and ADMET Predictions for 129 Compounds.

CMNPD ID	MW(g/mol)	Lipinski's rules	LogS	$LogD_{7.4}$	TPSA(Å²)	hERG(10μm)
CMNPD6532	363.42	0	-5.543	3.027	60.68	0.546
CMNPD18841	478.456	0	-5.852	2.891	132.13	0.287
CMNPD29188	510.634	0	-4.676	2.794	96.1	0.146
CMNPD18840	462.457	0	-5.388	2.976	111.9	0.275
CMNPD27687	510.634	0	-4.676	2.844	99.26	0.123
CMNPD27913	620.657	1	-9.892	3.860	111.63	0.115
CMNPD19652	373.459	0	-6.097	3.809	44.47	0.747
CMNPD27914	662.694	1	-10.323	4.011	128.7	0.114
CMNPD12678	546.759	0	-5.404	3.463	44.39	0.312
CMNPD22796	578.796	1	-8.743	5.388	34.14	0.824
CMNPD8410	394.602	0	-8.628	5.358	0	0.794
CMNPD22800	622.893	1	-8.950	5.480	29.6	0.832
CMNPD6529	338.41	0	-5.879	3.632	51.81	0.739
CMNPD29420	568.806	0	-4.019	3.240	84.41	0.050
CMNPD15979	566.79	0	-3.847	3.280	76.71	0.110
CMNPD12676	562.758	0	-4.861	3.182	61.46	0.456
CMNPD19653	332.403	0	-5.252	3.388	57.88	0.600
CMNPD6811	564.774	0	-4.718	3.271	75.62	0.213
CMNPD9231	444.656	0	-4.238	3.275	58.92	0.493
CMNPD6287	436.595	0	-7.397	4.355	18.46	0.785
CMNPD22799	620.877	1	-8.990	5.469	34.14	0.767
CMNPD6204	636.871	1	-6.374	4.099	95.89	0.534
CMNPD7175	630.825	0	-5.672	3.463	85.35	0.081
CMNPD9692	584.849	0	-3.866	3.152	65.97	0.109
CMNPD22798	608.866	1	-7.516	5.265	37.3	0.806
CMNPD25128	562.802	0	-5.095	3.594	44.39	0.242
CMNPD25797	544.556	0	-5.386	3.232	128.59	0.533
CMNPD3271	564.774	0	-4.410	3.072	72.46	0.278
CMNPD5448	548.775	0	-5.008	3.357	55.39	0.147
CMNPD17081	438.615	0	-4.296	3.235	25.36	0.746
CMNPD187	466.706	0	-5.856	3.789	60.69	0.708
CMNPD6203	534.825	0	-6.104	4.174	49.69	0.878
CMNPD11950	580.773	0	-4.324	2.974	92.69	0.285
CMNPD18887	649.913	0	-4.596	3.564	97.58	0.159
CMNPD16617	569.786	1	-6.645	4.153	71.55	0.722
CMNPD17082	440.631	0	-4.585	3.248	25.36	0.788
CMNPD22676	626.922	1	-7.312	4.434	47.92	0.760
CMNPD28415	476.617	0	-4.731	3.675	95.5	0.126
CMNPD9689	564.774	0	-4.718	3.129	75.62	0.157

CMNPD5593	384.56	0	-4.520	3.723	46.53	0.331
CMNPD6202	738.917	1	-7.141	4.429	142.09	0.149
CMNPD3766	446.72	0	-3.702	3.554	24.94	0.504
CMNPD21931	664.791	0	-5.674	3.709	89.34	0.503
CMNPD24198	438.608	0	-4.519	3.664	52.6	0.501
CMNPD28413	478.633	0	-5.076	3.954	95.5	0.091
CMNPD29417	583.22	0	-4.600	3.109	52.15	0.218
CMNPD3767	461.731	0	-5.231	4.180	41.93	0.470
CMNPD13935	384.395	0	-3.935	2.215	85.51	0.293
CMNPD16561	413.473	0	-6.654	3.829	75.63	0.512
CMNPD17083	456.63	0	-4.290	3.105	45.59	0.779
CMNPD22801	622.893	1	-8.284	5.658	29.6	0.766
CMNPD22876	454.61	0	-6.441	4.168	35.53	0.669
CMNPD24895	332.311	0	-4.129	2.736	68.29	0.380
CMNPD6258	344.451	0	-4.185	2.579	66.76	0.505
CMNPD24939	448.471	0	-4.061	2.476	91.29	0.243
CMNPD25312	402.575	0	-4.500	3.310	66.76	0.558
CMNPD18676	564.646	0	-3.273	1.950	117.85	0.163
CMNPD31192	624.906	1	-6.603	4.482	66.76	0.432
CMNPD31416	462.627	0	-4.464	2.550	99.38	0.348
CMNPD4409	410.642	0	-5.852	4.411	34.14	0.606
CMNPD6288	350.414	0	-4.030	2.757	69.92	0.898
CMNPD7826	396.615	0	-5.325	3.775	26.3	0.812
CMNPD10813	652.872	0	-5.052	3.748	118.22	0.225
CMNPD15432	488.665	0	-5.279	3.923	85.22	0.370
CMNPD16562	447.918	0	-7.037	3.853	75.63	0.590
CMNPD18523	588.917	1	-6.122	4.694	54.37	0.238
CMNPD22793	442.599	0	-5.434	4.606	46.53	0.572
CMNPD22794	456.626	0	-6.979	4.729	35.53	0.608
CMNPD23584	571.802	1	-5.727	4.218	82.55	0.500
CMNPD26179	517.589	0	-4.776	2.820	122.64	0.241
CMNPD31296	539.669	0	-4.148	1.738	99.21	0.186
CMNPD3986	493.644	0	-4.232	2.909	72.91	0.320
CMNPD3787	709.682	0	-4.729	3.546	140.83	0.135
CMNPD21352	656.904	1	-5.085	3.975	96.22	0.459
CMNPD9104	372.549	0	-4.187	3.324	54.37	0.069
CMNPD14874	410.598	0	-4.307	3.655	54.37	0.264
CMNPD17079	458.69	0	-5.409	3.909	25.36	0.754
CMNPD19255	406.61	0	-5.177	3.871	37.3	0.219
CMNPD21315	496.531	1	-3.540	1.673	188.8	0.734
CMNPD21727	452.591	0	-4.714	3.547	69.67	0.287
CMNPD23553	447.535	0	-3.962	2.448	83.8	0.158
CMNPD24892	332.311	0	-4.075	2.772	75.99	0.395

CMNPD27283	564.646	0	-3.240	1.937	117.85	0.189
CMNPD27665	364.309	0	-3.561	1.976	97.75	0.379
CMNPD28333	487.523	0	-4.036	2.816	124.48	0.670
CMNPD9167	481.633	0	-3.931	2.238	72.91	0.352
CMNPD4506	410.598	0	-4.556	3.099	35.53	0.743
CMNPD7823	412.614	0	-4.665	3.614	35.53	0.807
CMNPD29418	566.79	0	-3.724	2.967	70.53	0.105
CMNPD8781	664.766	0	-3.690	3.223	135.43	0.343
CMNPD1440	520.707	0	-4.429	3.029	105.45	0.168
CMNPD10150	364.309	0	-3.836	2.015	101.05	0.209
CMNPD11737	692.958	0	-4.726	3.592	106.81	0.213
CMNPD12052	464.738	0	-4.713	4.139	32.67	0.792
CMNPD12084	528.862	1	-7.745	5.403	49.69	0.480
CMNPD16065	460.611	0	-4.446	3.399	89.9	0.046
CMNPD16525	572.874	1	-8.647	5.724	46.53	0.362
CMNPD19749	403.438	0	-3.480	1.894	78.95	0.190
CMNPD19768	479.533	0	-3.899	2.410	117.2	0.176
CMNPD22201	564.646	0	-3.823	2.557	119.54	0.183
CMNPD22791	424.584	0	-6.998	4.550	34.14	0.648
CMNPD22797	624.865	1	-8.787	5.246	35.53	0.810
CMNPD25781	506.643	0	-3.865	2.641	103.78	0.017
CMNPD29424	666.215	1	-6.876	4.074	71.76	0.810
CMNPD30020	678.731	1	-4.873	2.935	181.44	0.518
CMNPD3987	493.644	0	-3.864	2.677	72.91	0.426
CMNPD6258	344.451	0	-4.185	2.579	66.76	0.505
CMNPD6260	358.434	0	-3.770	2.161	83.83	0.425
CMNPD8044	422.609	0	-4.919	3.796	38.69	0.461
CMNPD8376	677.88	0	-5.067	3.464	132.83	0.051
CMNPD1098	654.888	1	-6.912	4.417	77.38	0.799
CMNPD10398	725.681	1	-4.629	2.902	161.06	0.027
CMNPD12051	482.753	0	-4.601	3.685	49.54	0.621
CMNPD6262	428.657	0	-4.865	4.024	60.69	0.343
CMNPD14868	408.582	0	-4.991	3.382	51.21	0.318
CMNPD1423	446.672	0	-4.656	3.761	69.92	0.493
CMNPD17925	503.711	0	-3.648	2.175	67.01	0.286
CMNPD21065	538.608	0	-3.587	2.249	126.64	0.155
CMNPD2092	495.66	0	-3.883	2.976	72.91	0.343
CMNPD30029	560.555	0	-5.737	2.981	148.82	0.304
CMNPD31420	432.645	0	-5.325	4.254	69.92	0.269
CMNPD5748	424.625	0	-4.446	3.414	43.37	0.518
CMNPD3954	384.516	0	-4.291	3.673	48.06	0.178
CMNPD9792	460.611	0	-4.943	3.704	82.06	0.381
CMNPD11217	608.86	0	-4.624	3.452	107.22	0.039

CMNPD19934	707.949	0	-5.456	3.527	127.04	0.564
CMNPD12818	436.684	0	-4.857	4.196	55.12	0.732
CMNPD25333	424.717	0	-5.718	4.539	38.05	0.736
CMNPD13777	489.571	0	-4.200	3.173	84.16	0.792
Rilematovir [a]	500.090	0	-4.079	2.501	78.89	0.445
Sisunatovir [a]	446.170	0	-3.338	2.599	64.15	0.769

[a] Rilematovir and Sisunatovir are known RSV fusion (F) protein inhibitors and were used as positive controls.

Table S3. Raw MM/GBSA Data for 31 Compounds.

CMNPD ID	Bind	Coulomb	Covalent	Hbond	Lipo	Packing	SelfCont	Solv_GB	vdW	LE [a]
CMNPD6811	-111.15	-136.28	1.03	-0.03	-41.54	-8.62	0.00	125.08	-50.78	-2.65
CMNPD11950	-109.15	-120.62	5.74	-0.05	-43.29	-10.42	0.00	106.20	-46.70	-2.54
CMNPD3271	-108.29	-119.98	5.37	0.17	-46.28	-11.06	0.14	122.47	-59.13	-2.58
CMNPD29420	-106.94	-132.50	1.88	0.17	-42.45	-11.03	0.02	136.90	-59.93	-2.55
CMNPD15979	-104.85	-137.26	10.50	0.08	-49.61	-11.46	0.15	138.69	-55.94	-2.50
CMNPD9689	-102.39	-128.75	14.03	-0.44	-40.58	-12.63	0.04	131.14	-65.21	-2.44
CMNPD29418	-84.44	-19.20	2.58	1.24	-47.39	-15.45	0.15	57.92	-64.29	-2.01
CMNPD27283	-80.77	-52.41	11.13	0.76	-36.44	-7.33	0.00	69.97	-66.45	-1.92
CMNPD9692	-79.37	-77.58	7.63	0.34	-43.69	-5.48	0.04	99.78	-60.42	-1.85
CMNPD11737	-75.95	-4.47	10.26	-0.89	-40.20	0.42	0.06	24.49	-65.62	-1.52
CMNPD18887	-73.90	-36.81	7.76	-0.19	-40.43	1.33	-0.06	51.83	-57.33	-1.57
CMNPD24939	-73.87	-46.59	1.14	-0.34	-29.88	-9.48	0.01	56.07	-44.81	-2.24
CMNPD29188	-71.74	-39.00	5.71	-1.53	-31.40	-8.36	0.06	57.45	-54.67	-1.89
CMNPD28415	-70.22	-27.82	2.44	-0.22	-38.12	-1.85	-0.06	51.82	-56.41	-2.01

CMNPD22201	-66.28	-17.18	1.39	-0.20	-27.28	-7.17	0.02	38.18	-54.04	-1.58
CMNPD18676	-64.48	-41.78	7.71	-0.46	-35.45	-4.92	0.07	69.81	-59.46	-1.54
CMNPD31296	-63.59	-30.76	0.05	0.19	-32.17	0.79	0.04	53.99	-55.72	-1.63
CMNPD19749	-60.67	-32.21	3.79	0.84	-41.15	-5.52	0.01	65.78	-52.20	-2.02
CMNPD21727	-58.85	6.85	0.06	0.24	-35.68	0.77	0.01	15.24	-46.32	-1.78
CMNPD27687	-57.64	-35.98	4.91	0.98	-36.76	-5.20	-0.03	71.92	-57.49	-1.52
CMNPD13935	-57.63	-22.97	-1.36	1.24	-25.17	-6.49	0.00	47.31	-50.19	-1.99
CMNPD10150	-57.57	-59.61	2.67	-0.15	-35.53	-5.70	0.01	86.89	-46.16	-2.13
CMNPD23553	-56.51	-34.95	2.66	-0.32	-27.23	-8.58	-0.02	68.31	-56.38	-1.71
CMNPD16065	-55.89	-35.28	7.63	-0.43	-30.49	0.97	-0.06	42.40	-40.63	-1.69
CMNPD1440	-55.57	-42.71	0.15	0.54	-29.44	-0.38	0.03	60.23	-43.99	-1.50
CMNPD6811	-111.15	-136.28	1.03	-0.03	-41.54	-8.62	0.00	125.08	-50.78	-2.65
CMNPD11950	-109.15	-120.62	5.74	-0.05	-43.29	-10.42	0.00	106.20	-46.70	-2.54
CMNPD3271	-108.29	-119.98	5.37	0.17	-46.28	-11.06	0.14	122.47	-59.13	-2.58
CMNPD29420	-106.94	-132.50	1.88	0.17	-42.45	-11.03	0.02	136.90	-59.93	-2.55
CMNPD15979	-104.85	-137.26	10.50	0.08	-49.61	-11.46	0.15	138.69	-55.94	-2.50
CMNPD9689	-102.39	-128.75	14.03	-0.44	-40.58	-12.63	0.04	131.14	-65.21	-2.44
CMNPD29418	-84.44	-19.20	2.58	1.24	-47.39	-15.45	0.15	57.92	-64.29	-2.01
Rilematovir [b]	-33.38	-7.31	4.54	-0.28	-22.77	-8.39	54.01	-53.18	-1.01	-33.38
Sisunatovir [b]	-75.80	-113.05	0.34	-0.86	-36.22	-9.88	131.60	-47.74	-2.37	-75.80

[a] All energy values are reported in kcal/mol; Ligand Efficiency (LE) is unitless.

[b] Rilematovir and Sisunatovir are known RSV fusion (F) protein inhibitors and were used as positive controls.

Table S4. Key Interaction Distances for 11 Compounds.

CMNPD ID	Hydrogen Bonds [a]<br>AA-Distance H-A(Å)	Hydrogen Bonds<br>AA-Distance D-A(Å)	Hydrophobic Interactions<br>AA-Distance(Å)	π -Stacking<br>AA-Distance(Å)
CMNPD6811	Phe140C-3.55<br>Asp489B-3.02	Phe140C-4.09<br>Asp489B-3.73	Phe137A-3.60<br>Phe140B-3.49<br>Phe140C-3.62<br>Phe488B-3.47<br>Phe488C-3.95<br>Asp489B-3.94	Phe140B-4.68<br>Phe488A-4.39<br>Phe488C-3.62
CMNPD11950			Phe137B-3.35<br>Phe137C-3.59<br>Phe140A-3.48<br>Phe140B-3.89<br>Phe488A-3.35<br>Phe488B-3.50<br>Phe489A-3.84	Phe488B-3.87<br>Phe488C-4.48
CMNPD3271			Phe137C-3.58<br>Phe140B-3.52<br>Phe488A-3.38<br>Phe488B-3.32<br>Phe488C-3.33	Phe140A-4.47<br>Phe488B-4.21
CMNPD29420	Phe137C-2.35	Phe137C-2.95	Phe140B-3.55<br>Phe488A-3.55<br>Phe488B-3.49<br>Phe488C-3.60	Phe488A-4.90<br>Phe488B-5.36<br>Phe488C-3.60
CMNPD15979			Phe137A-3.51<br>Phe140B-3.48<br>Phe140C-3.66<br>Phe488B-3.79<br>Phe488C-3.56<br>Asp489B-3.74	Phe488A-4.62
CMNPD9689	Phe137B-2.81	Phe137B-3.64	Phe137C-3.73<br>Phe140A-3.96<br>Phe140B-3.65<br>Phe488A-3.57<br>Phe488C-3.50<br>Phe489C-3.62	Phe140C-4.60<br>Phe488A-3.75<br>Phe488B-4.54
CMNPD29418			Phe137A-3.87<br>Phe140A-3.75<br>Phe488A-3.52	Phe140B-4.79<br>Phe488C-3.88

			Phe488B-3.32<br>Phe488C-3.38	
CMNPD24939	Phe137B-3.24<br>Phe139B-2.64<br>Phe140B-3.30	Phe137B-4.01<br>Phe139B-3.33<br>Phe140B-3.94	Phe137B-3.59<br>Phe140B-3.70<br>Phe488B-3.57	Phe140B-5.16<br>Phe488A-5.26<br>Phe488C-3.73
CMNPD28415	Phe140C-2.04	Phe140C-2.56	Phe140B-3.09<br>Phe140C-3.77<br>Phe488A-3.06<br>Phe488B-3.24<br>Phe488C-3.33	
CMNPD19749			Phe140B-3.43<br>Phe140C-3.84<br>Phe488A-3.16<br>Phe488B-3.44<br>Phe488C-3.29	Phe488A-4.62
CMNPD10150	Asp486A-3.69<br>Phe488A-3.33<br>Phe488C-2.63<br>Asp489C-2.98	Asp486A-4.08<br>Phe488A-3.86<br>Phe488C-3.26<br>Asp489C-3.72	Phe140B-3.78<br>Phe488A-3.48<br>Phe488B-3.99<br>Phe488C-3.20	Phe488A-4.10

[a] Only the shortest distance is reported for residues with multiple interaction contacts.
